# Resurgence of Severe Challenging Behavior and Schedule Thinning with the Terminal Schedule Probe Method

**DOI:** 10.3390/bs15030382

**Published:** 2025-03-18

**Authors:** Craig W. Strohmeier, Elizabeth Thuman, John Michael Falligant, Mirela Cengher, Michelle D. Chin, Patricia F. Kurtz

**Affiliations:** 1 Kennedy Krieger Institute, 707 N. Broadway, Baltimore, MD 21205, USA; thuman@kennedykrieger.org (E.T.); chin@kennedykrieger.org (M.D.C.); kurtz@kennedykrieger.org (P.F.K.); 2 Department of Psychiatry and Behavioral Sciences, Johns Hopkins University School of Medicine, 733 N. Broadway, Baltimore, MD 21205, USA; 3 Department of Psychological Sciences, Auburn University, Auburn, AL 36849, USA; jmf0031@auburn.edu; 4 Department of Psychology, University of Maryland Baltimore County, 1000 Hilltop Circle, Baltimore, MD 21250, USA; cengher@umbc.edu

**Keywords:** functional analysis, multiple schedule, challenging behavior, resurgence, schedule thinning

## Abstract

Multiple schedules promote schedule thinning during treatment for challenging behavior. Some strategies for multiple-schedule-thinning progressions include dense-to-lean (DTL; gradually thinning schedules of reinforcement in small steps), fixed lean (FL; abruptly shifting to lean schedules), and terminal probe (TP; probing terminal schedule values to inform subsequent thinning steps) thinning. Recent research indicates that TP thinning offers an empirically derived process for schedule thinning to terminal schedule values. In the current investigation, we replicated, re-analyzed, and extended recent research on the TP-thinning method. The schedule-thinning outcomes were consistent with the initial investigation, indicating that longer periods of reinforcer unavailability were facilitated by TP thinning in comparison with DTL thinning. We also examined resurgence, or the temporary increase in challenging behavior after alternative behavior is placed on extinction, across a wide range of downshifts in reinforcement. When resurgence occurred across ranges of downshifts programmed for both schedule-thinning methods, it occurred more often with DTL thinning. The resurgence analyses did not reveal an exponential increase in challenging behavior as a function of downshifts in reinforcement, which is an interesting departure from recent investigations. Points of discussion include clinical implications and areas for future research on the TP-thinning method.

## 1. Introduction

Individuals with autism spectrum disorder (ASD) who demonstrate severe challenging behavior (e.g., self-injurious behavior [SIB], aggression, and disruptive behavior) are more likely to receive treatment with antipsychotic medication (Hill et al., 2014; Storch et al., 2012) and may be at greater risk for acute psychiatric hospitalization (Mandell, 2008). Additionally, challenging behavior may create barriers to accessing community and educational resources (Newcomb & Hagopian, 2018) and increased parenting stress (Kurtz et al., 2021). Therefore, assessing and intervening on challenging behaviors for individuals with ASD is critical for ensuring their long-term wellbeing and the safety of the individual and their families.

Effective treatment for challenging behavior begins with a pre-treatment functional analysis (e.g., Iwata et al., 1982/1994) to identify the relationship between challenging behavior and contextual variables (Heyvaert et al., 2014; Hurl et al., 2016). Recent reviews of functional analysis outcomes reported in the literature suggest that challenging behavior is often maintained by socially mediated reinforcers (i.e., functions), such as escape from demands, access to tangible items, or attention from others (Melanson & Fahmie, 2023). One common treatment for socially mediated challenging behavior includes a form of differential reinforcement of alternative behavior (DRA) that involves reinforcing a communicative response that serves the same function as challenging behavior (i.e., functional communication training [FCT]; Carr & Durand, 1985; Kurtz et al., 2011; Weber et al., 2024). Of note, FCT is considered a well-established treatment for challenging behavior demonstrated by children with ASD when implemented by clinicians, teachers, and parents (Kurtz et al., 2011; Muharib & Wood, 2018). FCT is often combined with extinction or punishment procedures to increase the treatment efficacy by ensuring a reduction in challenging behavior, in addition to increasing appropriate alternative behavior (Fisher et al., 1993; Hagopian et al., 1998; Rooker et al., 2013).

During the early stages of treatment with FCT, a dense schedule of reinforcement, such as a fixed ratio 1 (FR1), is necessary to establish and maintain alternative behavior in the form of functional communication responses (FCRs; Betz et al., 2013; Hanley et al., 2001). Once FCRs are established within an individual’s repertoire, the maintenance of a dense schedule of reinforcement is not practical for implementation in the natural environment. That is, caregivers and other intervention agents may find it difficult to reinforce FCRs consistently at a high rate, which may result in treatment integrity failures that compromise the effectiveness of treatment. Importantly, missed opportunities to reinforce alternative behavior may lead to the re-emergence of a child’s challenging behavior and subsequent relapse of undesirable caregiver behavior (i.e., provision of the functional reinforcer contingent on challenging behavior; Mitteer et al., 2018). Therefore, developing a feasible behavioral treatment requires thinning the schedule of reinforcement from continuous reinforcement of the alternative behavior to a leaner, more practical schedule (Hanley et al., 2001; Kranak & Brown, 2024; Tiger et al., 2008).

Multiple schedules, a type of compound reinforcement schedule, are commonly used to facilitate schedule thinning during the treatment of challenging behavior (e.g., Saini et al., 2016). Multiple schedules include two or more signaled, alternating schedules of reinforcement, whereby one signal (discriminative stimulus; S^D^) is most often correlated with an FR1 schedule for FCRs and extinction for challenging behavior, and the other signal (delta stimulus; S^∆^) is correlated with extinction for both the challenging behavior and FCRs. The schedule components alternate based on time, and schedule thinning typically occurs by increasing the duration of the S^∆^ component and decreasing, or holding constant, the S^D^ component (e.g., Hagopian et al., 2005). With a multiple-schedule treatment arrangement, individuals learn to emit responses differentially in accordance with the schedule components, which promotes discriminated responding and reduces excessive FCRs.

Hanley et al. (2001) introduced a tactic to thin multiple schedules during the treatment of challenging behavior, which is referred to as dense-to-lean (DTL) thinning. Using DTL thinning, a clinician initially introduces the delta stimulus component of a multiple schedule for a brief interval (e.g., 30 s). Schedule thinning proceeds by gradually extending the 30 s delta stimulus interval in small increments (e.g., 15–30 s) according to pre-determined progression criteria (e.g., three consecutive sessions where challenging behavior was observed at an 80% reduction relative to the baseline mean). Gradual increases in the duration are implemented to putatively mitigate escalations in challenging behavior related to increased time with programmed extinction for both the challenging behavior and FCRs. In the Hanley et al. investigation, schedules of reinforcement were effectively thinned to more practical levels, with no apparent disruption of the contingencies supporting alternative behavior. Challenging behavior also remained at clinically significant low levels for most of the treatment evaluation, but elevated rates of challenging behavior occurred when the terminal schedule value was introduced. Although the rates of challenging behavior ultimately decreased to clinically significant levels, the variable and elevated rates observed for the terminal schedule call into question the assumption that thinning reinforcement schedules in smaller steps effectively mitigates increases in challenging behavior as the reinforcement becomes lean. If challenging behavior is likely to occur during the terminal schedule regardless of the progression, it may be worth exploring alternative approaches to schedule thinning.

Hagopian et al. (2004) investigated the implementation of a fixed lean schedule of reinforcement by transitioning from a rich schedule of reinforcement immediately to a lean schedule of reinforcement for alternative behavior. The fixed lean schedule appeared to be more efficient (i.e., facilitating more rapid transitions to the terminal schedule) and effective at minimizing the total problem behavior throughout most of the schedule-thinning phase in comparison with DTL thinning. More recently, Betz et al. (2013) reported abruptly shifting to lean schedules of reinforcement using a multiple schedule arrangement. The investigators noted that establishing stimulus control over FCRs during dense schedule arrangements appeared to promote continued response differentiation of FCRs during more lean conditions (see Experiment 3). All four participants in the Betz et al. investigation demonstrated significant differentiation of FCRs across the S^D^ and S^∆^ components during the terminal schedule of reinforcement. Despite the encouraging data related to FCRs, 50% (2/4) of the participants did not demonstrate any instances of challenging behavior across the baseline (multiple or mixed 60 s [S^D^]/60 s [S^∆^] schedules) or terminal schedule (60/240 schedule) phases. The remaining two participants were noted as demonstrating minimal (i.e., “near zero”, pp. 235–236) rates of challenging behavior. Further research is needed in this area of applied research to clarify the procedures and expected challenging behavior outcomes during abrupt shifts to lean reinforcement conditions.

Hagopian et al. (2011) and, more recently, Kranak and Brown (2024) reviewed several approaches to schedule thinning, including a multiple-schedule-thinning strategy called terminal probe (TP) schedule thinning. Clinicians or researchers may proceed with TP schedule thinning by setting a priori criteria, such as a terminal treatment goal of an 8 min S^∆^ component that alternates with a 2 min S^D^ component of a multiple schedule. After setting the criteria, a probe session is conducted to determine the latency to challenging behavior after the onset of the delta stimulus component (Kranak & Falligant, 2022). Schedule thinning after the probe session involves starting with an S^∆^ component that is slightly briefer than the latency to challenging behavior observed during the probe. Progression and regression criteria may be used to determine when to proceed with thinning using a DTL strategy or when to probe the terminal schedule again. An advantage of the TP strategy is that the S^∆^ starting point is empirically derived based on the performance of the individual and may be closer to the terminal schedule than an arbitrary (e.g., 30 s) starting point.

One variation of TP thinning involves measuring the latency to clinically significant elevations (i.e., rates greater than 20% of the baseline mean) during the S^∆^ component of the TP session, rather than the latency to the first instance of challenging behavior, to derive an S^∆^ starting point (Strohmeier et al., 2024). Strohmeier et al. extended investigations of TP strategies by summarizing TP-thinning results from 24 applications of schedule thinning, during which clinicians measured the latency to clinically significant elevations in challenging behavior during a terminal schedule probe session. This method provided a criterion for establishing initial S^∆^ durations that yielded a more progressive S^∆^ starting point for multiple schedule thinning. The investigators also described a structured method for subsequent schedule-thinning progressions or further probing of terminal schedule values. Results of the investigation suggested that participants exposed to the TP-thinning method achieved leaner terminal schedules of reinforcement relative to participants who underwent more traditional DTL thinning. Although the use of this less conservative TP-thinning method appears to be a promising tactic to achieve more practical schedules of reinforcement, further replication of this specific TP-thinning method is necessary to increase the external validity of the procedures and outcomes.

Findings from the basic literature suggest that resurgence, which is considered the reemergence of a previously extinguished target behavior (e.g., challenging behavior) after an alternative behavior (e.g., FCR) is placed on extinction (Shahan & Sweeney, 2011), increases exponentially as a function of the size of the downshift in alternative reinforcement during schedule thinning (Shahan et al., 2020). Preliminary work in applied settings also suggests that this aspect of resurgence may be observed during the implementation of schedule thinning with multiple schedules (Shahan & Greer, 2021; Falligant et al., 2022). Shahan and Greer conducted a retrospective investigation of 10 applications of FCT schedule thinning to analyze the degree of resurgence of challenging behavior that occurred at varied magnitudes of downshifts in reinforcement. The study results were consistent with those from basic research, that is, larger magnitude downshifts in reinforcement led to a greater resurgence of challenging behavior. In other words, a larger magnitude downshift of reinforcement (e.g., 1 min to 8 min of active delta stimulus) yielded a greater resurgence of challenging behavior relative to a smaller magnitude downshift (e.g., 1 min to 2 min of active S^∆^). The TP-thinning method oftentimes involves larger magnitude downshifts than those previously reported in the literature. Therefore, analyzing the levels of resurgence at broad ranges of downshifts, including small and large magnitude downshifts that occur during TP thinning, may help to better characterize resurgence during schedule thinning. In turn, these findings may better prepare clinicians for varied presentations of resurgence when using different thinning strategies.

In the current paper, we report several replications of the TP-thinning method described by Strohmeier et al. (2024), with an updated cohort that comprised individuals with ASD admitted to an outpatient clinic for the assessment and treatment of severe challenging behavior. We also replicated the comparisons of outcomes from two groups of participants; one group who underwent TP thinning and another that underwent more traditional DTL thinning. Similar to Strohmeier et al., the results of the schedule-thinning outcomes were analyzed across groups using a retrospective consecutive controlled case series method (Hagopian, 2020). Finally, we extended the resurgence analyses from Strohmeier et al. by reporting resurgence in terms of the magnitude of resurgence in relation to the size of the downshift during schedule thinning.

## 2. Methods

### 2.1. Participants, Setting, and Analytic Design

The participants were 33 patients with intellectual disability and ASD who were admitted to an outpatient clinic for the assessment and treatment of severe challenging behavior and met the study inclusion criteria described below. The participants completed their course of clinical services and were discharged between 2012 and August 2023. The participants were admitted to the same day treatment and intensive outpatient programs described in Strohmeier et al. (2024, p. 678). A functional analysis using procedures described by Iwata et al. (1982/1994) was conducted with each participant, and functions of behavior were confirmed with structured criteria evaluation procedures, like those described by Hagopian et al. (2023). Additional functional analysis conditions conducted for some participants included escape from attention (Cengher et al., 2022), compliance with mands (Bowman et al., 1997), and interruption of free operant behavior (Hagopian et al., 2007). Based on the functional analysis outcomes, a treatment evaluation was conducted, and a multiple schedule (Saini et al., 2016) was used for schedule thinning. Behavior skills training (Ward-Horner & Sturmey, 2012) was used to train behavior therapists and caregivers to conduct sessions. The sessions took place in padded or unpadded therapy rooms (approximately 3 m by 3 m). Functional analysis and initial treatment sessions were 10 min, with some treatment sessions extended when necessary to simulate the natural environment. The overall session durations ranged from 10 to 30 min.

Data were analyzed using a retrospective consecutive controlled case series design (Hagopian, 2020) to identify consecutively encountered participants within the clinical database. The following criteria were established to identify participants: (a) the participant had a diagnosis of ASD; (b) a functional analysis that included at least three test and two control series was conducted using a multielement or pairwise design, and functional analysis results were confirmed using structured criteria (Hagopian et al., 1997, 2023; Roane et al., 2013); (c) a treatment using functional communication and extinction was evaluated using a reversal or multiple baseline design, and experimental control was observed via visual inspection; (d) a multiple schedule was used for schedule thinning, and a terminal schedule probe session was conducted; (e) independent observers collected data for a minimum of 20% of sessions across both the functional analysis and treatment evaluation to allow for the calculation of the interobserver agreement, with 80% or greater agreement; and (f) no supplemental components (e.g., competing stimuli, response cost, alternate activities, reinforcement for compliance during the S^∆^ for escape-maintained challenging behavior) were added to the treatment to facilitate schedule thinning. The inclusion criteria differed from Strohmeier et al. (2024) in two primary areas: first, the current investigation used a structured criteria evaluation to support the functional analysis conclusions. Structured criteria evaluation of the functional analysis results provided a more standardized set of procedures to increase replicability. Second, a more homogenous diagnostic profile was isolated for the current investigation (i.e., only individuals with autism were included). Provided all of the inclusion criteria were met, all consecutively encountered cases were retained for the investigation, regardless of the outcomes, to minimize the selection bias.

Based on a review of the clinical database, 201 participants had treatment evaluations that included TP sessions (described below). The first and fourth authors reviewed the identified cases for inclusion. In the event that there was a disagreement across cases, only those that achieved consensus agreement for inclusion were ultimately included in the investigation. Seven participants were retained from the dataset described by Strohmeier et al. (2024) since they met the inclusion criteria for the current investigation (i.e., documented diagnosis of ASD and functional analysis results confirmed with structured criteria evaluation; Cases 1, 2, 3, 4, 5, 6, 7). We identified 17 additional participants after screening the updated clinical records according to the inclusion criteria above (see Table 1). Overall, the 24 participants included 17 males and 7 females that ranged in age from 4 to 22 years (M = 11; see Table 1). Multiple applications per participant (Case 16) occurred when the TP session was implemented for multiple functions in a multiple baseline design, resulting in a total of 25 applications for the TP-thinning group.

An additional group of participants was identified to compare outcomes from more traditional DTL-thinning procedures with those obtained using TP-thinning procedures. Participants were identified for comparison using the same inclusion criteria as the TP group (described above), plus an additional criterion of using a multiple schedule and DTL thinning to facilitate schedule thinning without a TP session and derived progression. A reverse chronological search of the clinical database was used to initially identify 30 participants with DTL thinning, beginning with the year that the TP-thinning participants were admitted to and discharged from the outpatient programs. Due to the same reasons described above, we retained eight participants from Strohmeier et al. (2024) for the comparison group (Cases 1, 2, 3, 4, 5, 6, 7, 8). One additional participant was identified in the updated clinical records. In total, the comparison group consisted of nine participants who met the inclusion criteria (Table 2). Six males and three females, who ranged in age from 3 to 13 years (M = 9 years), were included. Multiple applications occurred when DTL thinning was implemented for multiple functions in a multiple baseline design (Cases 1 and 9) and across multiple caregivers (Case 1), resulting in a total of 13 applications for the DTL-thinning group.

### 2.2. Response Definitions

Individualized operational definitions for targeted challenging behaviors were generated based on a patient’s clinical targets. The most common forms of challenging behavior were SIB, aggression, and disruptive behavior. SIB was broadly defined as any behavior that caused or had the potential to cause harm to the person, including self-biting, hand-to-head hitting, self-scratching, and head banging. Aggression was broadly defined as any behavior that caused or had the potential to cause harm to others, including hitting, kicking, scratching, and throwing objects at other people. Disruptive behavior was broadly defined as any behavior that caused or had the potential to cause property damage or disrupt the environment, including banging on surfaces, throwing objects (not person-directed), ripping/breaking/tearing, and swiping objects off a table. Other behaviors included elopement, pica, vocal disruption (saying a profane word at or above conversational level), dangerous acts (e.g., standing on furniture, turning over furniture), fecal smearing, and licking.

### 2.3. Data Collection and Interobserver Agreement

Trained observers collected data on target responses with laptop computers to record the frequency of behavior, which was converted to a response rate (B-DataPro; Bullock et al., 2017). To calculate the interobserver agreement, session data were analyzed within 10 s intervals. For each interval, reliability coefficients were calculated based on the number of responses; specifically, within each interval, the lower number of responses was divided by the higher number and multiplied by 100. Interobserver agreement data were collected for 26.5% to 100% (M = 60.6%) of the functional analysis sessions for each participant. The IOA coefficients ranged from 96.5% to 100% (M = 98.6%) across the target behaviors for functional analyses. The data used to calculate the IOA were collected for 27.4% to 88.6% (M = 56.7%) of the treatment sessions; the coefficients ranged from 97% to 99.98% (M = 99.2%).

### 2.4. General Procedures

#### 2.4.1. Interpretation of Functional Analysis Data

The functional analysis results were confirmed using a structured criteria evaluation (e.g., Hagopian et al., 1997, 2023; Roane et al., 2013). To decrease the chance of human error, a customized spreadsheet that employed the criteria was used to perform the structured criteria evaluation for the individual functional analyses. The method involved identifying upper and lower criterion lines based on the control condition. Then, for each test condition, a quotient score was calculated, where the number of data points below the lower criterion line was subtracted from the number of data points above the upper criterion line and divided by the total number of data points in that condition. Quotient scores above 0.5 indicate differentiation. In some cases, quotient scores can be less than 0.5 but the criteria still indicate an identified function (e.g., if there is an upward trend in which all data points in the second half of the test condition are above the upper criterion line).

#### 2.4.2. Treatment

Data from the functional analysis served as the initial baseline sessions in the treatment evaluation (Falligant et al., 2020; Scheithauer et al., 2020). Procedures identical to the relevant test condition from the functional analysis were used in the return to baseline for the treatment evaluations with a reversal design. A continuous schedule of reinforcement (i.e., FR1) for alternative behavior (i.e., the FCR) and extinction for challenging behavior were used at the start of the treatment. In the event of uncontrollable or dangerous behavior, individualized session termination criteria were developed for all participants. Caregivers were involved in all aspects of the appointments throughout the assessment and treatment; caregivers’ preferences, as well as relevant clinical findings, guided the development of treatment goals, including the terminal S^Δ^ intervals.

A criterion for the challenging behavior percentage reduction was established at the onset of the treatment evaluation to guide the schedule thinning for each participant. Criteria for the progression of schedule thinning are often based on direct observation of the clinically significant reductions in challenging behavior for a pre-determined number of sessions (e.g., Hagopian et al., 2005). For the purposes of this investigation, a treatment reduction ≥ 80% from the baseline mean was considered clinically significant. An 80% reduction is a common clinical and research benchmark in the treatment of challenging behavior (e.g., Newcomb & Hagopian, 2018). Thus, the percentage reduction criterion was calculated by averaging the challenging behavior rates (responses per minute (rpm)) across the baseline sessions and multiplying by 0.20, resulting in an 80% reduction in rpm.

A multiple schedule was used for the schedule thinning, wherein two schedule components were alternated: when reinforcement was available, an S^D^ component in the form of the green side of a board was used, and when reinforcement was unavailable, an S^∆^ component in the form of the red side of a board was present. The red/green boards were either completely red on one side and green on the other, or half-green and half-red on the same side. A picture depicting the functional reinforcer for challenging behavior (FCR card) was placed on either the red or green areas of the board to indicate which component of the multiple schedule was active.

During the initial treatment sessions, only the S^D^ component was active, and all FCRs were reinforced. An initial brief S^∆^ or the terminal probe S^∆^ was introduced at the start of the schedule thinning. The progression criteria for schedule thinning included two sessions of clinically significant reductions in challenging behavior. If the challenging behavior reached clinically significant elevations across at least three sessions with an increasing trend, then the schedule would return to the previous schedule-thinning step.

### 2.5. Terminal Schedule Probe Thinning

TP thinning (Strohmeier et al., 2024) represents an empirically derived approach to schedule thinning that aims to integrate the caregiver’s terminal schedule goals with within-session evaluations of the challenging behavior during terminal schedule probe sessions. As part of the process, caregivers were asked to describe situations in which providing functional reinforcers for alternative behaviors might be difficult or impractical. For example, when treating a child with tangible-maintained challenging behavior, a caregiver may suggest that the tangible item should not be available for 50% of a 10 min session. The probe session would then be conducted with a multiple schedule that included a 5 min S^∆^. An S^∆^ of 8 min out of a 10 min session (80% of session) was suggested by the behavior therapist if the caregiver did not provide input on the terminal S^∆^ goal. The general goal of an 80% increase in S^∆^ duration was based on terminal schedule goals reported in previous applied research (e.g., Hanley et al., 2001). When the terminal schedule goal exceeded 10 min, clinicians collaborated with parents to transform the goal into a proportion (e.g., 30 min out of an hour is equivalent to 50% of the hour). The initial probe session then proceeded with the same proportional S^∆^ interval within the current session time (e.g., 5 min out of a 10 min session is equivalent to 50% of the session). Once the initial goal was met, the session times and S^∆^ intervals were proportionally extended until the parent’s goal was met.

In most cases, an initial brief S^∆^ (e.g., 30 s) was introduced after the rates of challenging behavior were below the 80% reduction criterion for at least two consecutive sessions. After low rates were observed in the sessions with the brief S^∆^, a terminal schedule probe session was conducted using the caregiver-informed S^∆^. During this session, the S^∆^ would take place between two S^D^ intervals. For example, if the S^∆^ was 8 min, the session would start with the S^D^ active for 1 min, followed by the 8 min S^∆^ being active, then the S^D^ was again active for 1 min. In the probe session, the latency to clinically significant elevations in challenging behavior were calculated after the onset of the S^∆^ (i.e., challenging behavior exceeded the reduction criteria). The latency to clinically significant elevations was used to surpass a potentially overly conservative S^∆^ derived from using the latency to a single instance of challenging behavior (e.g., Kranak & Falligant, 2022) and allow for maximal exposure to an S^∆^ interval that would facilitate extinction while potentially avoiding excessive elevations in challenging behavior. For example, if the challenging behavior exceeded a clinically significant elevation (i.e., 20% of the baseline mean), then the S^∆^ interval during the next session represented the latency to clinically significant elevation, rounded down to the nearest minute (see Strohmeier et al., 2024). After a schedule-thinning progression criterion was achieved, another probe session was conducted. In some cases, rather than returning to the probe, the S^∆^ was doubled until reaching 50% of the terminal schedule value, and then another probe was conducted (see, Strohmeier et al., 2024, pp. 680–681).

### 2.6. Dense-to-Lean Thinning

The therapists programmed FR1 schedules of reinforcement for the FCRs and extinction for challenging behaviors to evaluate the treatment with DTL thinning. Caregiver consultation informed the terminal schedule goals. After an initial brief S^∆^ (e.g., 15 s) was introduced, the progression of the duration of the S^∆^ interval was arbitrary, where they ranged from 5 s to 1 min increments.

### 2.7. Resurgence Analysis

The data were prepared and analyzed using procedures consistent with those described by Shahan and Greer (2021) and Falligant et al. (2022). Briefly, the response rates for challenging behavior were converted to a proportion of baseline for the first two sessions following a downshift in alternative reinforcement, as divided by the average rate of challenging behavior during the baseline. Resurgence was defined as an increase in the rates of challenging behavior during one of these two post-downshift sessions relative to the maximum rate of challenging behavior observed in the three sessions before the downshift in alternative reinforcement. We calculated the relative size of the downshift in alternative reinforcement availability based on the difference in the proportion of the session the S^D^ component of the multiple schedule was in operation for relative to the S^Δ^ component before and after each schedule-thinning step. We plotted the proportional levels of challenging behavior against the size of the downshift in alternative reinforcement in semi-logarithmic (ln) space to examine the relationship between resurgence and downshifts reinforcer availability during schedule thinning. Only applications in which resurgence was observed were included in the analysis. We subsequently fit a linear regression model, which was calculated using the ordinary least squares method, to these data. Because these data were in semi-logarithmic space, a significant linear trend would be evidence of an exponential relationship between the resurgence magnitude and downshift size.

In a supplemental analysis, we sought to define resurgence with reference to the loss of a clinically significant treatment effect. Given that an 80% reduction is a common clinical benchmark in the field of applied behavior analysis (e.g., Hagopian & Frank-Crawford, 2018), we defined clinically significant resurgence as occurring when resurgence was associated with the loss of at least an 80% reduction in challenging behavior observed prior to the downshift in the alternative reinforcement.

## 3. Results

### 3.1. Functional Analysis Outcomes

The functional analysis outcomes indicate that access to tangibles (19 out of 38 applications; 50%) was the most common function identified for intervention across both the TP-thinning and DTL-thinning groups. The second most common function was access to attention (11 out of 38 applications; 28.9%). Social negative functions (i.e., escape from demands and escape from attention) were identified for 5 out of 38 applications (13.2%). In two applications (5.3%), regained access to interrupted activities was identified (Hagopian et al., 2007), and there was one application (2.6%) in which a compliance with mands (Bowman et al., 1997) function was identified for intervention.

### 3.2. Treatment Evaluation

**Figure 1:**
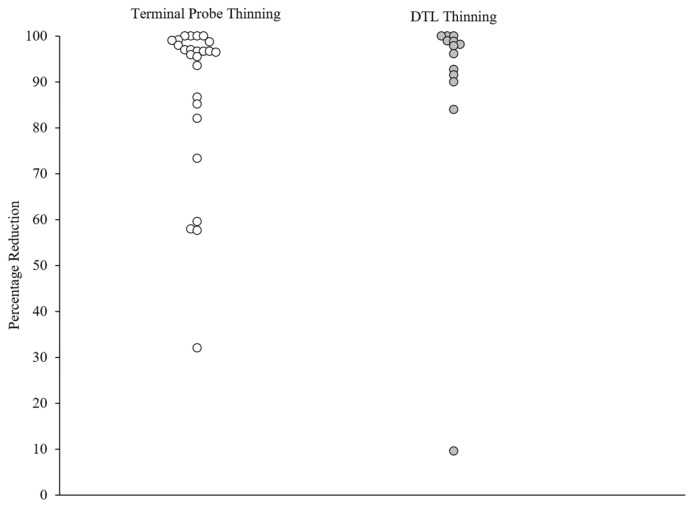
Percentage reduction in challenging behavior. Note: Overall treatment effects across schedule-thinning procedures. White circles represent applications of TP thinning, and dark gray circles represent applications of DTL thinning.

Treatments for all participants included FCT plus extinction using a multiple schedule for schedule thinning. Table 3 and Table 4 and Figure 1 present the final outcomes of the treatment evaluations after the schedule thinning. Clinically significant reductions in challenging behavior were observed for 20 out of 25 (80%) applications with TP thinning, and 12 out of 13 (92.3%) applications with DTL thinning. Across the five applications with FCT plus extinction with TP thinning, or the one application with DTL thinning, in which a clinically significant reduction in challenging behavior was not observed, there were no apparent differences in the behavioral profiles (e.g., function, topography). Clinically significant reductions in challenging behavior were observed in 32 out of 38 (84.2%) applications regardless of the thinning approach.

### 3.3. TP Thinning and DTL Thinning

Caregivers implemented sessions during the treatment evaluations for 22 out of 25 (88%; see Table 3) TP-thinning applications. In 15 out of 25 applications (60%) of TP thinning, the duration of the final S^∆^ matched the duration of the S^∆^ in the probe session, suggesting that in most cases, the caregiver-selected S^∆^ was achieved by the end of the treatment evaluation. In 10 of these applications, there was no further schedule thinning necessary after the probe session, indicating that an abrupt transition to lean schedule conditions did not produce any clinically significant elevation in challenging behavior that required regression to a schedule-thinning value that was active prior to the probe. In the other five cases, an individualized progressive thinning approach was implemented, where multiple steps followed the derived S^∆^ before reaching the probe value. In 5 out of 25 (20%) applications, the duration of the terminal S^∆^ surpassed the probe duration (Cases 2, 3, 7, 12, and 19); in three of these, the challenging behavior never met the criteria for schedule regression before reaching the terminal value. In the remaining 5 out of 25 (20%) applications, the duration of the final S^∆^ achieved was less than the duration of the TP session (Cases 4, 5, 11, 14, and 15). In 2 out of 25 (8%) applications, there was no brief initial S^∆^, and the schedule thinning began with the terminal schedule value (Cases 6 and 15).

The caregivers implemented sessions during the treatment evaluations for 6 out of 13 (46%; see Table 4) DTL-thinning applications. While the most common initial S^∆^ for the TP-thinning group was 30 s (88% of applications), the initial S^∆^ was less than 30 s in 10 out of 13 (76.9%) DTL-thinning applications. The duration of the final S^∆^ achieved was less than 5 min in 9 out of 13 (69.2%) DTL-thinning applications (range 10 s to 4 min).

**Figure 2:**
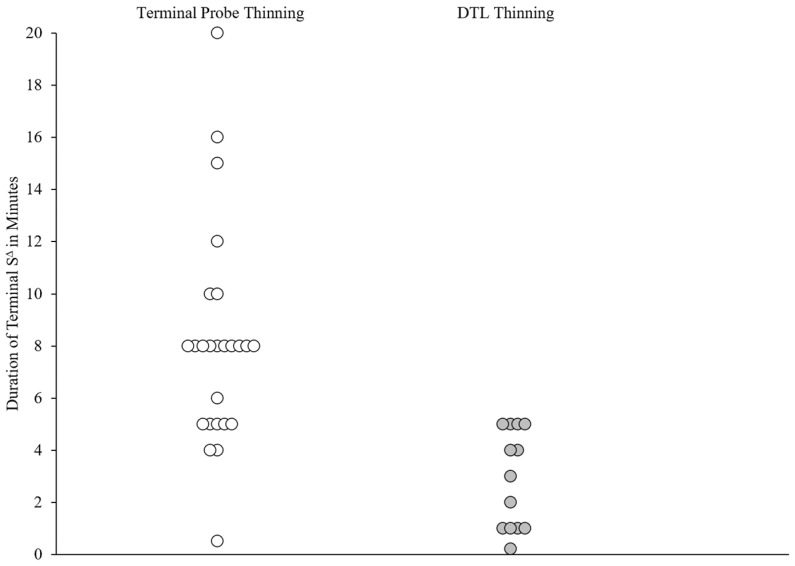
Duration of terminal S^∆^. Note: Terminal S^Δ^ durations across schedule-thinning procedures. White circles represent applications of TP thinning and dark gray circles represent applications of DTL thinning.

**Figure 3:**
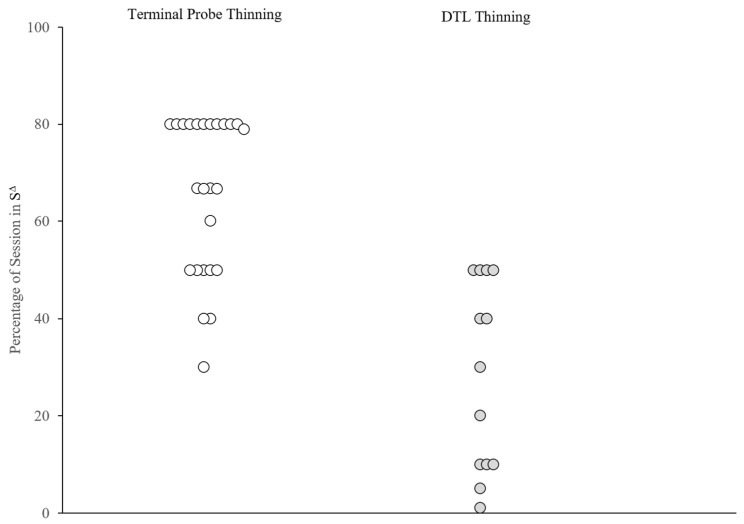
Percentage of session with active S^∆^. Note: Percentage of session with active S^Δ^ across schedule-thinning procedures. White circles represent applications of TP thinning and dark gray circles represent applications of DTL thinning. The Y–Axis denotes the percentage of session with an active S^Δ^ for the corresponding application.

Figure 2 and Figure 3 depict the schedule-thinning outcomes for the individual participants across the two groups. Figure 2 indicates that the majority (16 out of 25; 64%) of the TP-thinning applications reached a terminal S^∆^ of 8 min or more, whereas most applications in the DTL-thinning group (9 out of 13; 69%) reached a terminal S^∆^ that was less than 5 min. Figure 3 presents the proportion of session time with active S^∆^ contingencies. In 22 out of 25 (88%) TP-thinning applications, the percentage of sessions with an active S^∆^ was 50% or greater, whereas the percentage of sessions with an active S^∆^ was less than 50% in most (9 out of 13; 69%) DTL-thinning applications. The average number of sessions from the introduction of S^∆^ to the final S^∆^ interval was 21 (range = 6–69) for the TP-thinning group and 25 (range = 6–82) for the DTL-thinning group.

### 3.4. Resurgence Analysis Outcomes

**Figure 4:**
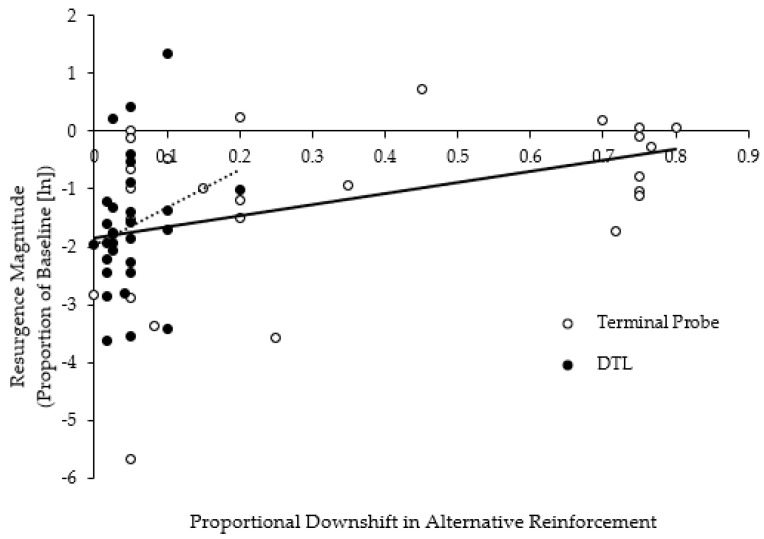
Proportional increase in the challenging behavior as a function of decreased alternative reinforcement across participants. Note: Data points indicate the logged proportion of baseline increase in challenging behavior across sessions that preceded and followed a decrease in the reinforcer availability. Data are displayed as a function of the proportional downshift in the reinforcer availability. Solid and dashed trend lines correspond to the ordinary least squares regression trendline for TP- and DTL-thinning applications, respectively.

Resurgence was identified for 15 out of 25 (60%) applications in the TP-thinning group, and 9 out of 13 (69%) applications in the DTL-thinning group. Figure 4 displays challenging behavior, depicted as a proportion of the baseline, across downshifts in the alternative reinforcement availability for the TP-thinning and DTL-thinning groups. The data are presented on a semi-logarithmic scale, where each data point represents a single FCT application (e.g., Shahan & Greer, 2021). Neither the TP-thinning group F(1,25) = 2.30, *p* = 0.14, nor the DTL-thinning group F(1,27) = 1.78, *p* = 0.19, showed an increase in challenging behavior as an exponential function of the downshift in the alternative reinforcer availability. 

**Figure 5:**
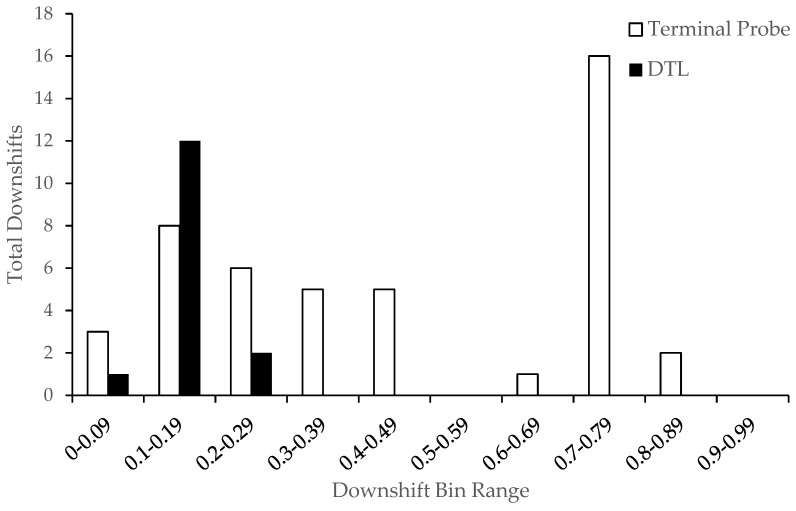
Total numbers of downshifts in reinforcer availability across downshift bin ranges. Note: Total number of downshifts in reinforcer availability at each downshift bin range. A downshift bin range denotes the proportional size of the change between the preceding and upcoming schedule-thinning step. For example, smaller bin ranges denote smaller proportional downshifts in the alternative reinforcement.

Downshifts in reinforcement were categorized into bin ranges based on the size of the downshift (Figure 5). Smaller bin ranges correspond to smaller proportional downshifts in alternative reinforcement. For example, increasing from 15 s of unavailability to 25 s of unavailability represents a proportional downshift of 0.2. In contrast, transitioning from 30 s of unavailability to 480 s represents a 0.75 proportional downshift. As expected, the TP-thinning method was associated with larger downshifts in reinforcement. The proportional downshift size for the TP-thinning group ranged from 0.00 to 0.89 (middle range = 0.40–0.49), whereas the downshift size was smaller for the DTL-thinning group and ranged from 0.00 to 0.29 (middle range = 0.10–0.19). Thus, despite the larger downshifts in the reinforcer availability, the TP-thinning method was not associated with a higher magnitude resurgence compared with the DTL-thinning method.

**Figure 6:**
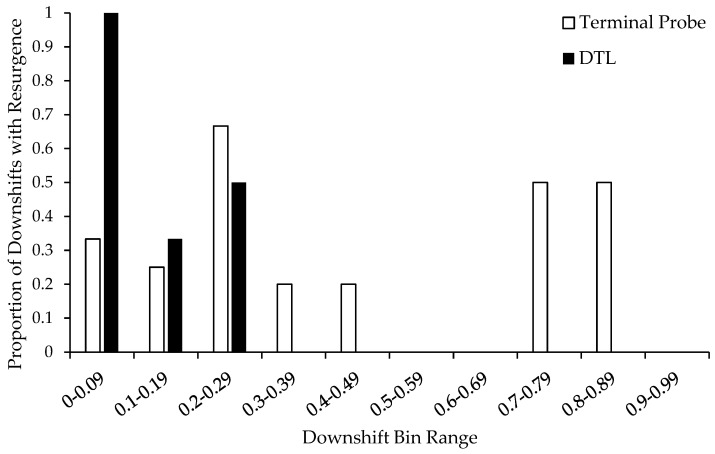
Total numbers of downshifts with resurgence across downshift bin ranges. Note: Proportion of downshifts (displayed in Figure 5) in which resurgence of problem behavior was observed across each downshift bin range.

Figure 6 shows the proportion of downshifts in which the resurgence of challenging behavior was observed. Overall, the resurgence of challenging behavior was equally likely to occur at smaller and larger downshift sizes for the TP-thinning group. Since the DTL-thinning group only had downshifts that were smaller (range 0.00–0.29), a comparison of the resurgence at larger downshifts could not be made between the groups. Nonetheless, both the TP- and DTL-thinning groups had downshifts within the 0.00–0.29 range. When resurgence did occur within these ranges, it was more likely for participants in the DTL-thinning group. Furthermore, at the smallest downshift size, 0.00–0.09, the resurgence of challenging behavior occurred for all the applications in the DTL-thinning group, in comparison with 33.33% of the TP-thinning applications at the same downshift size. We also evaluated the clinical significance of resurgence when it occurred at each downshift in reinforcement. When the resurgence occurred within the TP-thinning group, the challenging behavior exceeded 20% of the baseline mean (failed to meet an 80% reduction) during 26/36 (72%) downshifts. In the DTL-thinning group, the challenging behavior reached clinically significant elevations during 21/30 downshifts (70%). Therefore, clinically significant resurgence occurred at similar overall levels during the schedule thinning, regardless of the method.

## 4. Discussion

In the current investigation, we compared schedule-thinning outcomes from two groups of participants: one group who underwent traditional DTL schedule thinning, and another that underwent more abrupt TP schedule thinning. The results were analyzed within and between the groups of participants using a retrospective consecutive controlled case series method. On average, both the TP- and DTL-thinning groups achieved effective treatment outcomes (average of 80% reduction in severe challenging behavior; Figure 1). The average percentage reduction was slightly higher in the DTL-thinning group (12/13; 92.3%) in comparison with the TP-thinning group (20/25; 80%). Nonetheless, visual inspection suggests that only a minority of applications in both groups failed to meet an 80% reduction, and the difference between the two groups did not rise to the level of statistical significance (χ^2^(1) = 0.975, *p* = 0.323). The TP-thinning method yielded longer periods of reinforcer unavailability in comparison with DTL thinning (see Figure 2 and Figure 3). As a group, most applications of the TP-thinning method (16/25; 64%) exceeded an 8 min S^∆^, whereas the applications of DTL thinning resulted in a maximum S^Δ^ duration of 5 min (4/13; 30%). These results suggest that the TP-thinning method is effective at producing a longer terminal S^∆^ while maintaining clinically significant reductions in challenging behavior. For parents of a child with severe challenging behavior, this means reassurance in resuming the activities matched to the terminal S^∆^ duration without resultant distress that severe behavior may escalate to clinically significant levels. One major limitation of the current investigation was the absence of a direct comparison of schedule thinning across the TP- and DTL-thinning methods when similar terminal S^∆^ durations were selected by the caregivers. In the future, a prospective study should arrange for matched terminal S^∆^ durations across both methods so that more definitive data can be generated regarding the effectiveness and efficiency of schedule-thinning methods. An additional limitation was the lack of treatment integrity data for the applications of both DTL and TP thinning. These limitations were largely inherent to the retrospective and clinical source of our data. Future prospective investigations should ensure randomized assignment to schedule thinning groups with matched terminal schedules, as well as documentation of that schedule-thinning methods are followed as prescribed.

Consistent with Strohmeier et al. (2024), the results of the current investigation indicate that TP schedule-thinning methods facilitate rapid transitions to practical terminal S^Δ^ durations. Interventions that include longer periods of time for caregivers to engage in activities other than monitoring the delivery of the functional reinforcer for their child has potential to both increase the feasibility of treatment implementation and increase social validity. Saini et al. (2016) emphasized the importance of matching S^Δ^ intervals to the requirements of an individual’s natural environment. Although a 5 min S^Δ^ interval may suffice to facilitate functional reinforcer unavailability for some brief activities (e.g., child completing a small chore, caregiver answering a brief phone call), caregivers would likely benefit from treatments that offer the flexibility to extend an S^Δ^ duration when needed. Therefore, the TP-thinning method may be useful for programming more flexible treatment planning by exploring the boundaries of terminal reinforcement schedule possibilities based on caregiver input. Planning flexibly for the durations of functional reinforcer availability and unavailability can help prepare both the child and caregiver for instances where the terminal schedule goal may require reasonable variation (e.g., a chore takes longer than the typical time, or an important conversation that diverts a caregiver’s attention [and precludes delivery of the functional reinforcer] extends past 5 min).

Although the current investigation data appear promising, this study did not include a characterization of FCR patterns during terminal schedule probe sessions or throughout the treatment evaluation. Some recent investigations of multiple schedule thinning report specific strategies related to first establishing the stimulus control of FCRs across the S^D^/S^∆^ components of the multiple schedule, prior to abruptly shifting to lean schedules of reinforcement (Betz et al., 2013; Fisher et al., 2015). Betz et al. (2013) investigated the patterns of stimulus control over FCRs across S^D^ and S^∆^ multiple schedule components prior to abruptly shifting to a lean schedule of reinforcement. The investigators found that stimulus control over FCRs was maintained during the abrupt dense-to-lean shifts. Future investigations of TP thinning should borrow the FCR measurement and analytic strategies described by Betz et al. and Fisher et al. to determine whether multiple schedule arrangements continue to exert stimulus control over FCRs during TP thinning. The literature is overall sparse regarding abruptly shifting to fixed lean multiple schedule arrangements. Even more limited is research that adequately describes the processes responsible for the simultaneous maintenance of stimulus control over FCRs and reductions in severe challenging behavior. This area of applied research will benefit from the continued investigation of schedule-thinning strategies that arrange for more abrupt shifts to lean schedules of reinforcement, and the advancement of clinical schedule-thinning tactics that maintain alternative behavior while reducing severe challenging behavior.

Maintaining therapeutic gains and preventing a return of challenging behavior during the schedule-thinning process is both an important clinical consideration and a topic that has received attention from applied, basic, and translational researchers that investigate resurgence (Greer & Shahan, 2019). Strohmeier et al. (2024) reported observing resurgence in only 13 of 24 (54.17%) applications of TP thinning, whereas resurgence occurred in 16 of 18 (88.89%) applications of DTL thinning. In the current investigation, resurgence was observed in 15/25 (60%) applications of TP thinning and 9/13 (69%) applications of DTL thinning. Although the differences in proportions of applications with resurgence across methods in the current study were not as robust as the findings of Strohmeier et al., resurgence still occurred less often with TP thinning despite the larger downshifts in reinforcer availability. These findings may suggest that the TP-thinning procedures facilitate the mitigation of resurgence. Nonetheless, a prospective investigation with a more even distribution of participants across groups is necessary to increase the internal and external validities of these findings. Strohmeier et al. also analyzed the magnitude of resurgence following downshifts in reinforcer availability for the first through fifth sessions post-downshift in reinforcer availability. Comparisons of the mean resurgence magnitude across DTL and TP thinning showed a slightly higher resurgence magnitude in the TP-thinning group, but the differences were not statistically significant. In the current investigation, we analyzed the relationship between resurgence and downshifts in the reinforcer availability during schedule thinning. Interestingly, we did not observe an exponential relationship between the magnitude of resurgence and downshifts in the alternative reinforcement (Figure 4). In the DTL-thinning group, this absence may be attributed to the relatively small reductions in reinforcer availability during thinning steps (Figure 5). The lack of an exponential relationship in the TP-thinning group is more intriguing, given the large and abrupt downshifts in reinforcement, and may reflect the behavior-altering effects of the initial terminal schedule probe session and subsequent thinning procedures.

Greer et al. (2024) alluded to a potential “zone” of reinforcer unavailability (p. 15) wherein the schedule of reinforcement can be thinned without evoking clinically significant rates of challenging behavior. The TP schedule-thinning method endeavors to provide a clinically informed, optimal zone of reinforcer unavailability that will facilitate schedule thinning by (1) introducing an initial abrupt shift to the terminal schedule value, (2) deriving the latency to a clinically significant rate of challenging behavior (i.e., greater than 20% of baseline mean) within the S^∆^ interval, and (3) using this value to program subsequent schedule-thinning progressions. A single exposure to the terminal schedule during the probe session may provide sufficient contact with extinction contingencies to enhance the discriminative control of the S^∆^ and facilitate further schedule thinning. This enhanced discriminative control, combined with an empirically derived S^∆^, could facilitate reduced levels of resurgence during subsequent thinning steps, allowing the thinning process to progress within an optimal zone. Such a mechanism would support the use of TP methods for expediting reinforcement schedule thinning. Some evidence of this process may be preliminarily captured in the data depicted in Figure 6. The direct comparison of resurgence between the two schedule-thinning methods was possible in the 0.0–0.29 downshift bin range. Within this limited range of comparison, resurgence was observed more often during the applications of DTL thinning (during which no terminal schedule probe session occurred). While these preliminary findings are encouraging, the limited comparisons of bin ranges should temper any far-reaching conclusions regarding the relationship between the TP session and resurgence. A related limitation of these findings is the retrospective nature of the investigation. This limitation highlights the need for further investigation with prospectively identified and randomly assigned participants to explore methods of identifying optimal schedule-thinning zones that facilitate processes related to the extinction of target and alternate behaviors, as well as discriminable contingencies.

Recently, Podlesnik et al. (2024) encouraged investigators to examine varied criteria to characterize resurgence. Given the clinical nature of our investigation, as a supplemental analysis, we conducted an additional evaluation of the prevalence of resurgence based on a more conservative clinically derived relapse criterion. While many applied studies define resurgence as an increase in behavior post-downshift relative to the maximum rate of behavior pre-downshift (e.g., Briggs et al., 2018; Kranak & Falligant, 2022; Muething et al., 2021), this definition may not capture or reflect the clinical significance (or lack thereof) of the putative resurgence effects. For example, a slight increase in challenging behavior (e.g., a rate of behavior increase from 2 rpm to 2.1 rpm) pre-to-post downshift may meet the technical definition of resurgence but not be a cause for clinical concern. In the current investigation, when resurgence occurred in either condition, it generally reached clinically significant levels. This finding provides a degree of support for the clinical relevance of investigating the resurgence of challenging behavior with the commonly used definition. The replication of these results is necessary to support the generality of our findings.

Future research should explore the behavior-altering effects of terminal probes, particularly their role in reducing resurgence and enhancing the discriminative control of severe challenging behavior. Additionally, the arrangement and interspersal of specific downshifts in reinforcement availability should be systematically examined to determine their impact on the efficacy and efficiency of schedule thinning. In the literature, and based on our own clinical observation, multiple schedule components may be represented by different colors (e.g., red and green, white and black), arranged on opposite sides of the same board, on two different boards, or partitioned across the same side of a board. Additionally, the programming of schedule components may vary within and across investigation sites (e.g., rapidity and frequency of schedule component alternation, duration of schedule components, and visual presentation of the S^D^ and S^∆^ components; see Saini et al., 2016, p. 434). Finally, FCR topographies may vary according to the needs of the individual and environment. Clarifying subtle procedural differences may help to account for certain instances of failure to replicate investigation outcomes and isolate the specific variables related to the schedule-thinning efficiency and effectiveness. Increasing the implementation consistency of multiple schedule-thinning tactics across clinical practices and investigation sites will ensure that the most efficacious and effective procedures are implemented, as well as the most valid investigation outcomes.

In addition to the replication and extension of Strohmeier et al. (2024), the current study adds further support to the outcomes described by Hagopian et al. (2004) and, more recently, Chesbrough et al. (2024), supporting the notion that abrupt transitions to the terminal schedule may promote more efficient and effective schedule-thinning approaches relative to the traditional methods characterized by gradual reductions in the reinforcer availability. This study specifically highlighted the clinical application of a terminal schedule probe method to inform multiple-schedule S^Δ^ intervals during the treatment of severe challenging behavior in an outpatient setting. Establishing an effective and empirically derived schedule-thinning process is essential for optimizing the treatment efficiency. By minimizing unnecessary thinning steps, this approach reduces the risk of prolonged treatment evaluations and maximizes the time spent at terminal reinforcement schedules, ultimately improving the feasibility and outcomes. Ultimately, this represents a promising area for reverse-translational work, which could provide a deeper understanding of the mechanisms underlying effective schedule thinning and inform the development of more robust clinical protocols. That is, first determining when and where resurgence may occur in the natural environment. Re-creating such conditions in more controlled settings may facilitate the evaluation of procedures that maintain treatment effects in the most ecologically valid contexts.

## Figures and Tables

**Table 1:** TP-thinning participant demographics.

Case	Age	Sex	Level of Functioning
1	14	M	Mild
2	12	M	Mild
3	19	F	Severe
4	14	M	Moderate
5	14	F	Mild
6	12	M	Severe
7	9	F	Mild
8	14	M	Severe
9	11	M	Severe
10	15	M	Severity unspecified
11	11	F	Moderate
12	4	M	Severe
13	11	F	Average
14	7	F	Severe
15	8	M	Severe
16	13	M	Severe
17	9	M	Severe
18	8	M	Average
19	22	M	Severe
20	17	F	Moderate
21	5	M	Developmental delay
22	6	M	Moderate
23	13	M	Moderate
24	11	M	Mild

Note: M—male; F—female. The level of functioning was gathered from the documentation provided at the time of the intake evaluation.

**Table 2:** DTL-thinning participant demographics.

Case	Age	Sex	Level of Functioning
1	9	M	Moderate
2	9	M	Moderate
3	8	F	Moderate
4	8	M	Moderate
5	10	M	Moderate
6	13	M	Mild
7	8	M	Severe
8	13	F	Moderate
9	3	F	Developmental delay

Note: M—male; F—female. The level of functioning was gathered from the documentation provided at the time of the intake evaluation.

**Table 3:** Treatment and schedule-thinning information for TP-thinning participants.

Case	Application	Treatment	Duration of Initial S^Δ^	Terminal Probe Duration	Derived S^Δ^	Duration of Final S^Δ^ Achieved	Percentage of Session in S^Δ^	Percentage Reduction	Resurgence
1	1	Escape from Attn-Caregiver	30 s	8 min	8 min	8 min	80%	100%	N
2	2	Mands-Caregiver	15 s	8 min	8 min	20 min *	67%	100%	Y
3	3	Attention-Caregiver	30 s	8 min	8 min	16 min *	80%	96.39%	N
4	4	Interruption-Caregiver	30 s	8 min	50 s	5 min	50%	96.94%	Y
5	5	Escape	30 s	15 min	15 min	8 min	67%	96.91%	Y
6	6	Escape-Caregiver	8 min	8 min	3 min 30 s	8 min	80%	99.01%	N
7	7	Attention-Caregivers	30 s	8 min	8 min	10 min *	80%	93.56%	Y
8	8	Tangible-Caregiver	30 s	8 min	8 min	8 min	80%	100%	N
9	9	Tangible	30 s	5 min	5 min	5 min	50%	32%	Y
10	10	Tangible-Caregivers	30 s	8 min	8 min	8 min	80%	97.95%	Y
11	11	Tangible-Caregiver	30 s	3 min	2 min 30 s	30 s	30%	82.05%	Y
12	12	Escape-Caregivers	30 s	5 min	5 min	8 min *	80%	96.67%	N
13	13	Divided Attn-Caregivers	30 s	5min	5 min	5 min	50%	98.63%	Y
14	14	Tangible	30 s	8 min	4 min	4 min	40%	73.33%	Y
15	15	Tangible-Caregivers	8 min	8 min	1 min	6 min	60%	57.58%	Y
16	16	Tangible-Caregivers	30 s	5 min	1 min	5 min	50%	95.45%	Y
16	17	Divided Attn-Caregivers	30 s	5 min	5 min	5 min	50%	59.57%	N
17	18	Tangible-Caregivers	30 s	8 min	50 s	8 min	80%	96.67%	N
18	19	Interruption-Caregivers	30 s	8 min	8 min	15 min *	78.95%	86.67%	Y
19	20	Tangible-Caregiver	30 s	8 min	1 min	8 min	80%	96.67%	Y
20	21	Tangible-Caregiver	30 s	12 min	7 min	12 min	80%	99.07%	N
21	22	Tangible-Caregiver	30 s	4 min	4 min	4 min	40%	95.85%	Y
22	23	Tangible-Caregivers	30 s	8 min	7 min	8 min	80%	100%	Y
23	24	Attention-Caregivers	30 s	8 min	8 min	8 min	80%	85.11%	N
24	25	Divided Attn-Caregivers	30 s	10 min	10 min	10 min	66.67%	57.89%	N

Note: Attn—attention; S^Δ^—delta stimulus; N—no; Y—yes. * Denotes applications for which the duration of the final S^Δ^ achieved progressed further than the initial terminal probe duration.

**Table 4:** Treatment and schedule-thinning information for DTL-thinning participants.

Case	Application	Treatment	Duration of Initial S^Δ^	Duration of Final S^Δ^ Achieved	Percentage of Session in S^Δ^	Percentage Reduction	Resurgence
1	1	Tangible-Caregiver	15 s	2 min	20%	92.68%	Y
1	2	Attention-Caregiver	15 s	1 min	10%	90%	Y
1	3	Tangible-Caregiver	15 s	1 min	10%	98.78%	Y
1	4	Attention-Caregiver	15 s	1 min	10%	98.96%	N
2	5	Tangible Edible	15 s	4 min	40%	96.08%	Y
3	6	Divided Attn	15 s	3 min	30%	9.52%	Y
4	7	Attention	5 s	5 min	50%	100%	Y
5	8	Escape from Attn	30 s	4 min	40%	84%	Y
6	9	Tangible	15 s	5 min	50%	91.52%	N
7	10	Tangible	1 min 30 s	5 min	50%	100%	Y
8	11	Tangible	30 s	5 min	50%	98.13%	Y
9	12	Tangible-Caregiver	5 s	30 s	5%	100%	N
9	13	Attention-Caregiver	5 s	10 s	5%	97.83%	N

Note: Attn—attention; S^Δ^—delta stimulus; Y—yes; N—no.

## Data Availability

Data available upon reasonable request from the corresponding author.
